# Exploring Genomic Variants Related to Residual Feed Intake in Local and Commercial Chickens by Whole Genomic Resequencing

**DOI:** 10.3390/genes9020057

**Published:** 2018-01-24

**Authors:** Jie Liu, Ranran Liu, Jie Wang, Yonghong Zhang, Siyuan Xing, Maiqing Zheng, Huanxian Cui, Qinghe Li, Peng Li, Xiaoyan Cui, Wei Li, Guiping Zhao, Jie Wen

**Affiliations:** 1 Institute of Animal Sciences, Chinese Academy of Agricultural Sciences, Beijing 100193, China; liujie84130@163.com (J.L.); liuranran@caas.cn (R.L.); wangjie4007@126.com (J.W.); yhzhang1981@163.com (Y.Z.); xingsiyuan@caas.cn (S.X.); zhengmaiqing@caas.cn (M.Z.); cuihuanxian@caas.cn (H.C.); liqinghe@caas.cn (Q.L.); lipengnd@126.com (P.L.); cxyan813@163.com (X.C.); liweirfi@126.com (W.L.); 2 State Key Laboratory of Animal Nutrition, Beijing 100193, China; 3 College of Animal Science, Jilin University, Changchun 130062, China

**Keywords:** genome resequencing, residual feed intake, genomic variants, biomarkers, chickens

## Abstract

Improving feed efficiency is a major goal in poultry production to reduce production costs and increase profitability. The genomic variants and possible molecular mechanisms responsible for residual feed intake (RFI) in chickens, however, remain poorly understood. In this study, using both local and commercial breeds, genome re-sequencing of low RFI and high RFI chickens was performed to elucidate the genomic variants underlying RFI. Results showed that 8,505,214 and 8,479,041 single nucleotide polymorphisms (SNPs) were detected in low and high RFI Beijing-You chickens, respectively; 8,352,008 and 8,372,769 SNPs were detected in low- and high-RFI Cobb chickens, respectively. Through a series of filtering processes, 3746 candidate SNPs assigned to 1137 genes in Beijing-You chickens and 575 candidate SNPs (448 genes) in Cobb chickens were found. The validation of the selected 191 SNPs showed that 46 SNPs were significantly associated with the RFI in an independent population of 779 Cobb chickens, suggesting that the method of screening associated SNPs with whole genome sequencing (WGS) strategy was reasonable. Functions annotation of RFI-related genes indicated that genes in Beijing-You were enriched in lipid and carbohydrate metabolism, as well as the phosphatase and tensin homolog (PTEN) signaling pathway. In Cobb, however, RFI-related genes were enriched in the feed behavior process and cAMP responsive element binding protein (CREB) signaling pathway. For both breeds, organismal development physiological processes were enriched. Correspondingly, *NOS1*, *PHKG1*, *NEU3* and *PIP5K1B* were differentially expressed in Beijing-You, while *CDC42*, *CSK*, *PIK3R3*, *CAMK4* and *PLCB4* were differentially expressed in Cobb, suggesting that these might be key genes that contribute to RFI. The results of the present study identified numerous novel SNPs for RFI, which provide candidate biomarkers for use in the genetic selection for RFI. The study has improved knowledge of the genomic variants and potential biological pathways underlying RFI in chickens.

## 1. Introduction

As with most animal production systems, the cost of feed in chicken meat production is a high proportion of the total farming expense, being nearly 70% of the total cost of poultry production [1]. The selection of more efficient animals reduces production costs, decreases the area of land required for feed production and reduces environmental impact of nitrogen and phosphorus excretion resulting from the digestion/metabolic processes. 

Residual feed intake (RFI), defined as the difference between the actual feed intake (FI) and predicted requirements based on growth and maintenance, was first proposed as an alternate measure of feed efficiency (FE) by Koch et al. [2]. RFI is a sensitive and accurate indicator that is increasingly accepted as an alternative measure of FE of livestock. RFI has a moderate heritability (0.21 to 0.49) in broilers and responds to selection [3]. Elucidation of the genomic variants for RFI could potentially be important for marker- or gene-assisted selection [4].

Current whole genomic sequencing methodologies are important tools to unravel the mechanisms of complex traits, facilitate new understanding of the genetic regulation of phenotype and allow the identification of potential biomarkers for early or more accurate genetic prediction [5]. In recent years, some progress has been gained in attempts to identify the molecular mechanisms underlying RFI. Transcriptome analysis of mRNA and microRNA in skeletal muscle in pigs showed that genes involved in mitochondrial energy metabolism were downregulated, while those involved in skeletal muscle differentiation and proliferation were upregulated in low RFI (LRFI), as compared to high RFI (HRFI) animals [6]. The duodenal transcriptome architecture of extreme RFI phenotypes of six brown-egg dwarf hens showed that significant differentially-expressed genes were involved in several specific biological functions related to the processes of digestibility, metabolism, biosynthesis and energy homeostasis [7]. Combination analysis of genome-wide association and transcriptome sequencing in chickens showed that the effective single-nucleotide polymorphisms (SNPs) related to energy utilization were located in a 1-Mb region (16.3–17.3 Mb) of chicken chromosome 12, and liver transcriptomes may contribute to RFI, which is directly influenced by appetite, cell activities and fat metabolism [8]. Genome-level analyses of genetic markers and candidate genes for RFI in pigs by genome-wide associations and systems genetic analyses revealed that the SNPs of *MAP3K5*, *PEX7* and *DSCAM* might be interesting markers for RFI. Functional annotation of genes indicated that regulation of protein and lipid metabolism, gap junction formation, inositol phosphate metabolism and the insulin signaling pathway were significant biological processes and pathways associated with RFI [4]. Genome-wide association analysis of FI and RFI in Nellore cattle identified several markers located on chromosomes 4, 8, 14 and 21 in regions near genes regulating appetite and ion transport [9]. 

There are more than one hundred domestic chicken breeds available across China, and collectively, they have roughly the same market share in China as do introduced commercial lines. Beijing-You chickens, one of the representative domestic chickens, are known for their peculiar characteristics including higher intramuscular fat content and chewy texture [10], which are appealing palatability factors for Chinese consumers. The Cobb line has been intensely selected for high feed efficiency and growth rate and is an important commercial breed world-wide. To gain insights into the molecular alterations underlying differences between HRFI and LRFI chickens, whole genome sequencing (WGS) was conducted using HRFI and LRFI birds of local and commercial breeds. 

## 2. Materials and Methods

### 2.1. Ethics Statement

All of the animal experiments were conducted in accordance with the Guidelines for Experimental Animals established by the Ministry of Science and Technology (Beijing, China). Animal experiments were approved by the Science Research Department (in charge of animal welfare issue) of the Institute of Animal Sciences, Chinese Academy of Agricultural Sciences (IAS-CAAS) (Beijing, China). Ethical approval on animal survival was given by the animal ethics committee of IAS-CAAS (No. IASCAAS-AE-03). 

### 2.2. Animals and Sample Collection

A population of 400 Beijing-You chickens (200 males and 200 females) with a similar body weight, aged 70 days, was selected from the base population of the Institute of Animal Sciences (CAAS), and these birds came from 75 male and 153 female families (Table 1). Upon receipt, all chickens were housed in individual stair-step cages for FE measurement. The birds were individually weighed at the beginning (Day 70, BW_70_) and end of the FE test (Day 98, BW_98_) and were fed *ad libitum* with daily measurement of FI for the intervening 28 days (Days 70–98 comprised the rapid growth phase for Beijing-You). The diet was based on the Feeding Standard for Chickens established by the Ministry of Agriculture (Beijing, China). For the 28-day period, FI (FI_70–98_), weight gain (WG_70–98_) and RFI (RFI_70–98_), as well as the metabolic BW (BW^0.75^) at 70 days were calculated. The corresponding data for 220 male Cobb chickens from 64 male families (Table 1) were measured for the rapid growth phase (Days 28–42) of Cobb, developmentally equivalent “finisher” stages, by Guangdong Xinguang Nongmu Co., Ltd. (Foshan, China). RFI was calculated as:

RFI = FI − (b_0_ + b_1_BW^0.75^ + b_2_WG)

where b_0_ is the intercept and b_1_ and b_2_ are partial regression coefficients of FI on BW^0.75^ and WG, respectively. RFI values were generated by the regression procedure using SAS 8.0 for Windows statistical software (SAS Institute, Cary, NC, USA). 

Genomic DNA was isolated from whole blood samples collected from 48 HRFI and 48 LRFI chickens (the details of the group information are shown in Table 1), within each breed, using the phenol-chloroform method. DNA quality was determined by gel electrophoresis, and the 48 individual DNA samples from each group were used to construct three pools, each of 16 samples. For Beijing-You chickens, each pool consisted of 8 males and 8 females. In brief, there were three pools and 16 chickens of each pool for each sub-group. Paired-end sequencing libraries were constructed using the Nextera DNA Library Preparation Kit (Illumina Inc., San Diego, CA, USA) according to the manufacturer’s standard protocol. At the end of the feeding studies (Day 98), birds were killed, and weight percentages of breast and thigh muscles from the two groups (*n* = 48, HRFI and LRFI, in Beijing-You chickens) were measured by standard dissection procedures. Breast muscle and liver samples from subsamples (*n* = 8) of HRFI and LRFI male Beijing-You chickens were cut into small pieces, rapidly snap-frozen and stored at −80 °C for quantitative real-time PCR (qRT-PCR) analyses. Likewise, samples of breast muscle and hypothalamus from HRFI and LRFI Cobb chickens (each *n* = 8) were collected and saved the same way. 

### 2.3. Genome Sequence Assembly and Data Analysis

All libraries were sequenced on the Hiseq 2500 platform (Illumina) instrument with 125-bp paired-end reads. Reads of extremely low quality (>10 consecutive nucleotides with Phred scores < 10), with adaptor contamination or without a quality control-passed paired read were discarded using NGSQC toolkit (v2.3.3), resulting in a qualified clean dataset. More than 20× coverages were determined for each pool (Table 1). All clean sequenced reads were mapped to a reference genome (Galgal 4.0) using the BWA tool (v0.7.10) [11] with default parameters. Output SAM files were converted to BAM files with command Samtools (v0.1.1.18), and all converted BAM files were sorted with command Samtools (v0.1.1.18). PCR duplications were removed with the “rmdup” argument in Samtools (v0.1.18) [12] after mapping. SNPs were identified and genotyped for each sample pool with the mpileup function in Samtools (v0.1.18), as well as the VarScan.jar tool (v2.3.7) written in Java [13]. Only highly confident variants supported by both methods were used for further downstream analyses.

The sequence data reported in this paper have been deposited in the Genome Sequence Archive of BIG Data Center, Beijing Institute of Genomics (BIG), Chinese Academy of Sciences and is publicly accessible at http://gsa.big.ac.cn/preview/preview.action?code=WkQSr8M0 (accession no. PRJCA000311).

### 2.4. SNP Detection and Function Annotation

To identify SNPs with frequencies that consistently differed across replicates (pools) of these divergent populations, the divergence of allelic frequency between two phenotypically-extreme groups in the two breeds of chickens was statistically tested using analysis of variance and F-tests. SNPs with Benjamini-adjusted *p-*values of *<*0.05 were analyzed further. Pairwise fixation index (F_ST_) values were calculated using the F_ST_-test tool in the PoPoolation2 package. SNPs with F_ST_ values in the top 5% and allelic frequency changes greater than 35% (greater than the median of allelic frequency divergence in the top 5% SNPs) were identified as trait-related SNPs. Genes harboring these SNPs (50 kb of upstream and downstream of the SNPs) were selected for pathway and function enrichment analyses using the Ingenuity Pathway Analysis (IPA) system (http://www.ingenuity.com) according to the default parameters. Functional and canonical pathway analyses were used to identify significant biological functions and pathways; associated networks were also constructed.

### 2.5. SNP Validation by Sanger Sequencing

Nine randomly-chosen SNPs were subjected to validation by PCR analysis and Sanger sequencing using 16 phenotypically-divergent chickens. Targeted sequences were first PCR-amplified using 50 ng of genomic DNA with a Taq PCR MasterMix (Tiangen Biotech Co., Ltd., Beijing, China). Verification of PCR was performed by 1% agarose gel electrophoresis. Amplicons were then purified according to the EasyPure PCR Purification Kit’s standard protocol (Transgen Biotech Co., Ltd., Beijing, China) and sent to Tianyi Huiyuan (Beijing, China) for Sanger sequencing using primers described in Table S1. Results were analyzed using DNAMAN 8.0 (Lynnon Biosoft, San Ramon, CA, USA) (http://www.lynnon.com/), and ratios of bases occurring at SNP locations were recorded.

### 2.6. Association Analysis of the SNPs from Whole Genome Sequencing in the Validation Population of Cobb

In the validation population, there were 779 Cobbs (345 males and 434 females), which were the offspring of the generation for WGS bird screening. The RFIs of these chickens were measured by the methods described above. One hundred ninety SNPs were randomly selected from the candidate SNPs obtained from the WGS of Cobb. Genotyping was done on Axiom^®^ SNP arrays using the Affymetrix GeneTitan^®^ system according to the procedure described by Axiom 2.0 Target Prep 384 Samples Protocol (Affymetrix; Santa Clara, CA, USA) (https://www.thermofisher.com/). Genetic analyses were performed using ASREML [14] packages under the R v3.1.1 environment [15]. Breeding values of RFI were estimated using the BLUP based on an animal model with a relationship matrix. The models used in matrix notation were as follows:

*y* = Xb + Za + e

where *y* is the vector of observations; b, the vector of fixed effects of sex; a, the vector of random direct genetic effects; e, the vector of random residual effects; X and Z are incidence matrices relating the observations to the respective fixed and direct genetic effects.

The association analyses of estimated breeding values (EBVs) and SNPs were conducted using the Wald test under the R v3.1.1 environment [15]. The associations between SNP marker genotypes and RFI were estimated by using Proc GLM in SAS 9.4 (SAS Institute Inc., Cary, NC, USA). Additive genetic effects were estimated by pair-wise comparison of the two homozygous genotypes, and the dominance effects were calculated as the deviation of the heterozygote effect from the average of the two homozygous genotypes.

### 2.7. Quantitative Reverse Transcription-PCR Validation of Expression of Candidate Genes

To investigate whether the expression profiles of the variant genes differed between HRFI and LRFI groups, key genes (Table S2) that were enriched in the canonical pathways were chosen for qRT-PCR analyses. Total RNA was extracted from hypothalamus, breast muscle and liver using the RNAsimple Total RNA Kit (Tiangen) according to the manufacturer’s recommendations. For complementary DNA (cDNA) synthesis, 1 μg of total RNA was reversed transcribed with high capacity cDNA reverse transcription kits according to the manufacturer’s protocol (TaKaRa, Dalian, China). Zero-point-five microliters of cDNA serving as a template in a 20-μL PCR mixture were subsequently used for qRT-PCR analyses with an ABI 7500 Detection System (Applied Biosystems, Foster City, CA, USA) and primers designed using Primer Premier 5.0 software (PREMIER Biosoft, Palo Alto, CA, USA), as listed in Table S2. Eight independent replications were used for each assay. The mRNA abundance of candidate genes was determined using the KAPA SYBR^®^ FAST qPCR Master Mix (2×) Universal Cocktail (KAPA Biosystems, Boston, MA, USA). A corrected Ct (ΔCt) was calculated by subtracting the β-actin Ct value from that of the target genes for each sample. Relative differences from the control sample (the HRFI groups) were then calculated according to the 2^−ΔΔCT^ method. Differences between the two groups were analyzed using Student’s *t*-test for independent samples in SAS 8.0 for Windows.

## 3. Results

### 3.1. Characteristics of Birds in High and Low Phenotypic Groups

A summary of the phenotypic measurements of Beijing-You and Cobb chickens is presented in Table 2. There was no significant difference between HRFI and LRFI sub-groups within breeds in the initial body weights (BW_70_ of Beijing-You and BW_28_ of Cobb) and the final BW (BW_98_ of Beijing-You and BW_42_ of Cobb), nor the average daily gain (ADG). Notice, as expected, how different the means are for the Beijing-You and Cobb chickens. There were, however, significant differences (*p* < 0.01) in daily feed intake (DFI) between the phenotypically-divergent groups in both breeds. Consequently, the differences in mean RFI values and the feed conversion ratio (FCR) between LRFI and HRFI chickens in each breed were highly significant (*p* < 0.01). 

### 3.2. Genomic Variants Related to Residual Feed Intake in Beijing-You Chickens

**Figure 1:**
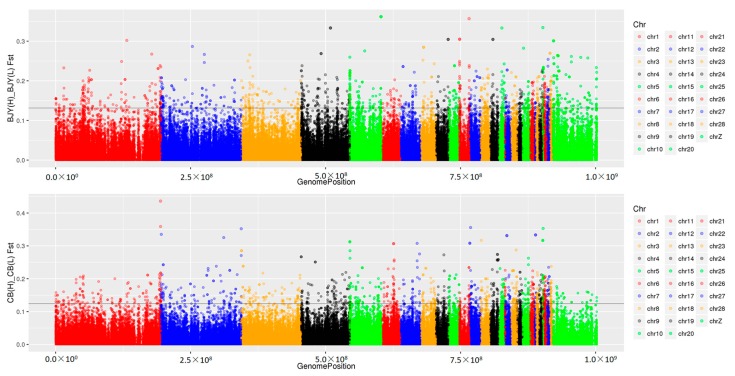
Distribution of SNPs with F_ST_ values in the top 5% in Beijing-You (Upper) and Cobb (Lower). More significant SNPs were enriched on *Gallus gallus* chromosome 1 (GGA1), GGA2, GGA4 and GGA Z in Beijing-You, while more significant SNPs were enriched on GGA1, GGA2, GGA3, GGA4 and GGA Z in Cobb.

**Figure 2:**
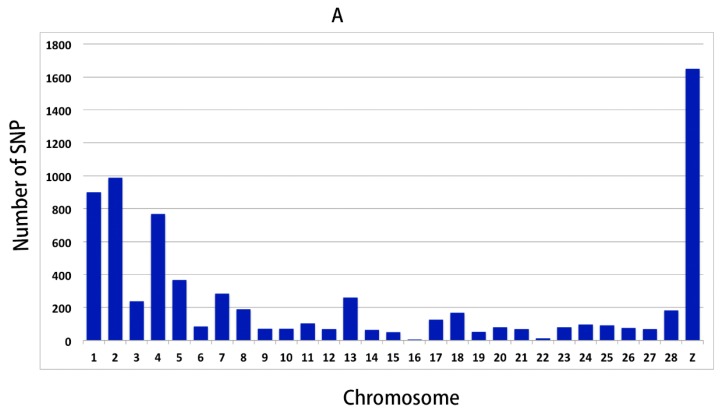

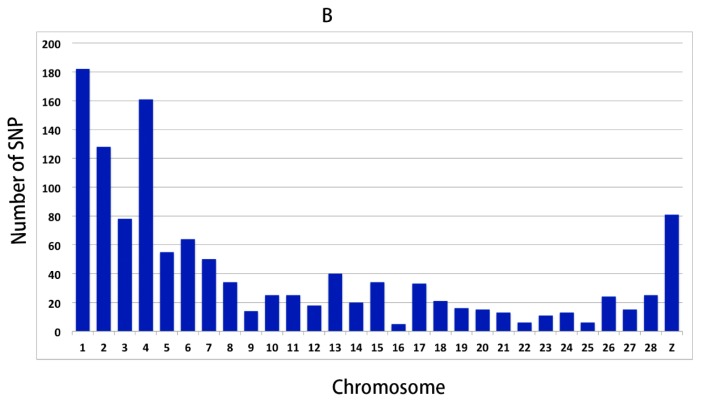
Distribution of SNPs obtained after filtering across chromosomes. There were 6288 and 1001 SNPs in Beijing-You (**A**) and Cobb (**B**) breeds, respectively.

**Figure 3:**
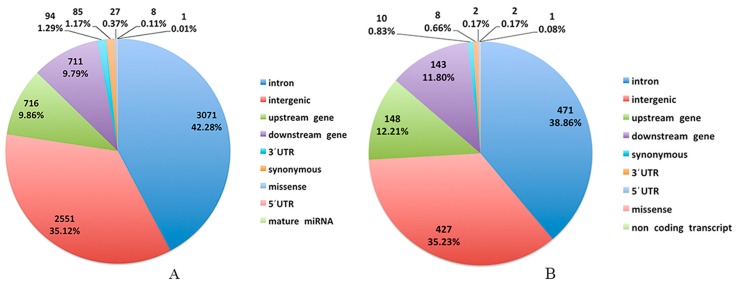
Categorization of the candidate SNPs in Beijing-You (**A**) and Cobb (**B**) chickens.

In LRFI and HRFI Beijing-You chickens, 8,505,214 and 8,479,041 SNPs were detected by applying an advanced detection algorithm and filtering criteria (Table S3). There were 26,125 SNPs with F_ST_ values in the top 5% shown in Figure 1. More significant SNPs were enriched on chicken (*Gallus gallus*) chromosomes (GGA) 1, GGA2, GGA4 and GGA Z. After filtering with allelic frequency divergence >35% between the HRFI and LRFI groups, there were 6288 SNPs identified as candidates for RFI traits (Figure 2A, Table S4). Consistently, over 4000 SNPs were found on four chromosomes GGA 1, GGA2, GGA4 and GGA Z. Among them, 2542 SNPs were located in intergenic regions and 3746 in genes, including upstream and downstream genes. The SNPs with synonymous and missense mutation were only 85 and 27 (Figure 3A). The 3746 SNPs were then annotated to 1137 genes (Table S4). 

**Figure 4:**
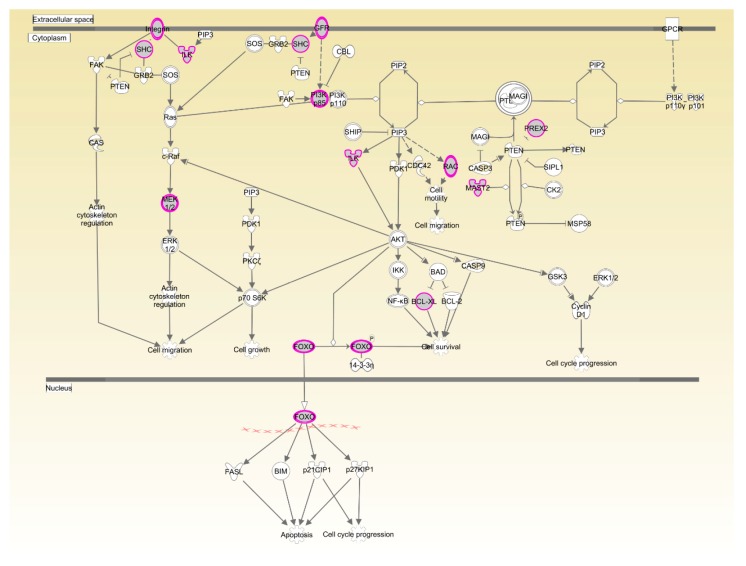
Phosphatase and tensin homolog (PTEN) signaling pathway enriched in Beijing-You chickens. Pink symbols show the genes found in the current study, while white symbols indicate genes that are functionally associated.

**Figure 5:**
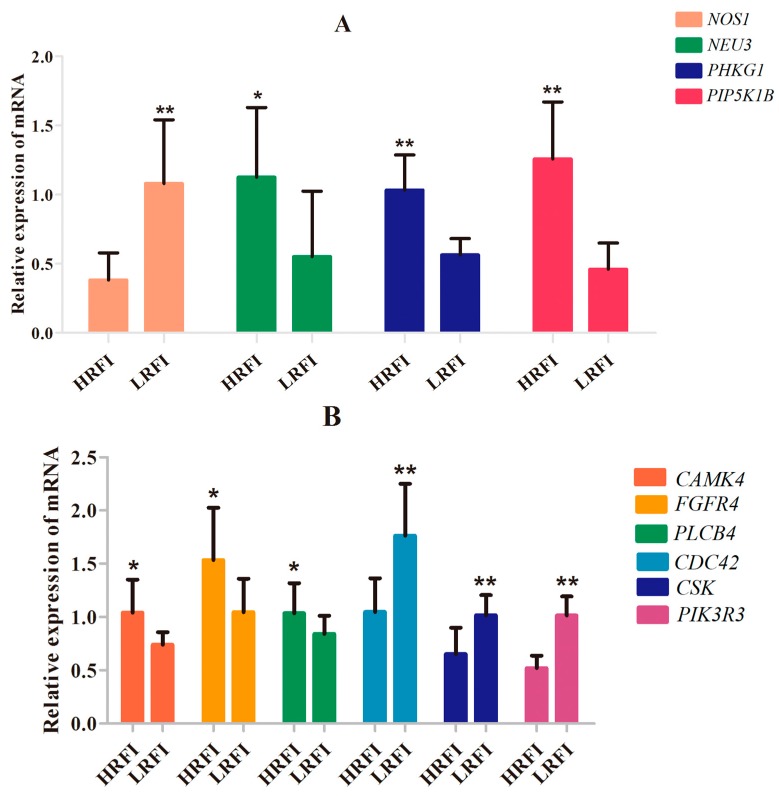
The expression level of genes related to enriched canonical pathways. (**A**) The differentially-expressed genes related to nNOS signaling in skeletal muscle (*NOS1*) and PTEN signaling, as well as lipid and carbohydrate metabolism in liver (*NEU3*, *PHKG1*, *PIP5K1B*) of Beijing-You chickens. (**B**) The differentially expressed genes related to cAMP responsive element binding protein (CREB) signaling in the hypothalamus (*CAMK4*, *FGFR4*, *PLCB4*) and genes related to Rac signaling and actin cytoskeleton signaling in the breast muscle (*CDC42*, *CSK*, *PIK3R3*) of Cobb chickens. Data are means ± standard deviation (SD) (*n* = 8 per group). Statistical significance was calculated by Student’s *t*-test; * *p* < 0.05, ** *p* < 0.01.

Functional annotation and pathway analysis were performed using IPA. Specific for Beijing-You, phosphatase and tensin homolog (PTEN) signaling, as well as lipid and carbohydrate metabolism were enriched (Figure 4, Table 4, Figure S1). Correspondingly, hepatic abundance of *NEU3*, *PHKG1* and *PIP5K1B* transcripts that participate in lipid and carbohydrate metabolism were upregulated (*p* < 0.05) in the HRFI compared to the LRFI birds (Figure 5A). 

### 3.3. Genomic Variants Related to Residual Feed Intake in Cobb Chickens

In Cobb chickens, 8,352,008 and 8,372,769 SNPs were detected in the LRFI and HRFI sub-groups (Table S3). There were 15,350 SNPs with F_ST_ values in the top 5% shown in Figure 1. More significant SNPs were enriched on GGA1, GGA2, GGA3 and GGA4. With the same data filtering criteria as for Beijing-You chickens, 1001 SNPs were obtained with >600 on GGA 1, GGA2, GGA3, GGA4 and GGA Z (Figure 2B, Table S5). Of these, 575 SNPs were located within genes, including 10 synonymous mutations and two missense mutations (Figure 3B). The 575 SNPs were then annotated to 448 genes (Table S5). The number of SNPs identified in Beijing-You was more than twice that of Cobb. 

**Figure 6:**
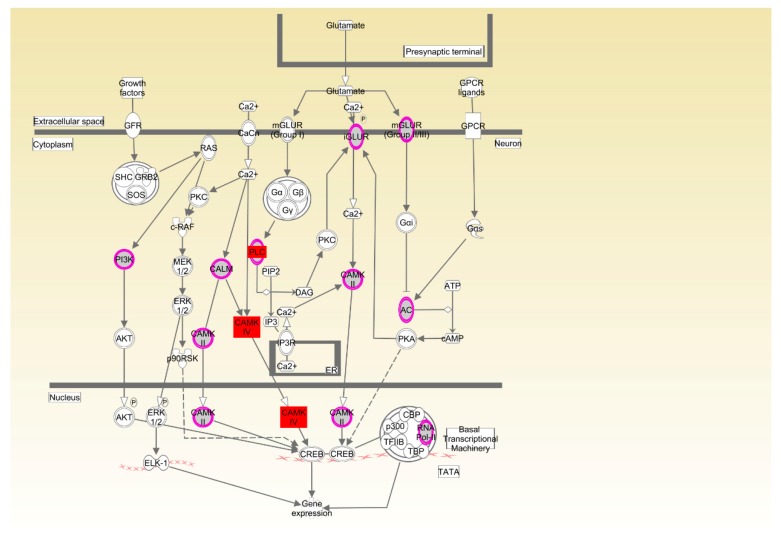
CREB signaling enriched in neurons in Cobb chickens. Pink symbols show the genes found in the current study, while white symbols indicate genes that are functionally associated. The red symbols show the genes that were differentially expressed in hypothalamus.

Specific for Cobb, function annotation showed that genes were enriched in behavior-related physiological process (Table 3). Feed behavior played a critical role in feed intake, and variation in feed intake was associated with variation in RFI. cAMP responsive element binding protein (CREB) signaling is a key factor in the control of appetite and feed intake by the hypothalamus. CREB signaling in neurons as the canonical pathway was significantly enriched in Cobb chickens (Figure 6, Table 4). Correspondingly, the expression levels of *CAMK4*, *PLCB4* and *FGFR4* in the hypothalamus were higher in HRFI chickens than in LRFI chickens (*p* < 0.05, Figure 5B). 

### 3.4. Common Features of Both Breeds

**Figure 7:**
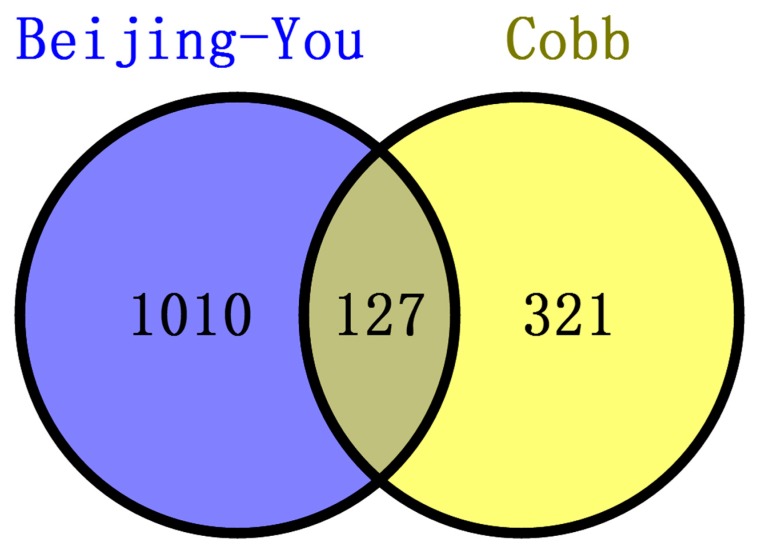
Numbers of genes near significant SNPs associated with RFI in Beijing-You and Cobb. There were 1137 genes and 448 genes identified in Beijing-You and Cobb, respectively, of which 127 were commonly found in both breeds.

Only 127 enriched genes were common to the two breeds (Figure 7). There were, however, several physiological processes commonly enriched in the two breeds, such as the development of nervous system, connective tissue and the skeletal and muscular systems (Table 3).

**Figure 8:**
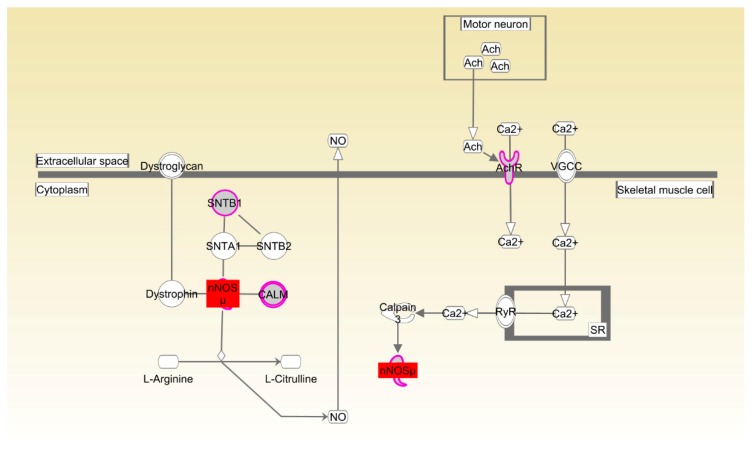
nNOS signaling in the skeletal muscle cell pathway enriched in Beijing-You chickens. Molecular interaction and symbols are the same as the description in Figure 6. The red symbols show the genes that were differentially expressed in breast muscle.

In Beijing-You, the enrichment of nNOS signaling in skeletal muscle cells as the significant pathway supported the important role played by organismal development for the RFI trait (Figure 8, Table 4). Consistently, the abundance of *NOS1* transcripts was upregulated (*p* < 0.01) in the breast muscle of LRFI chickens compared to HRFI chickens (Figure 5A).

**Figure 9:**
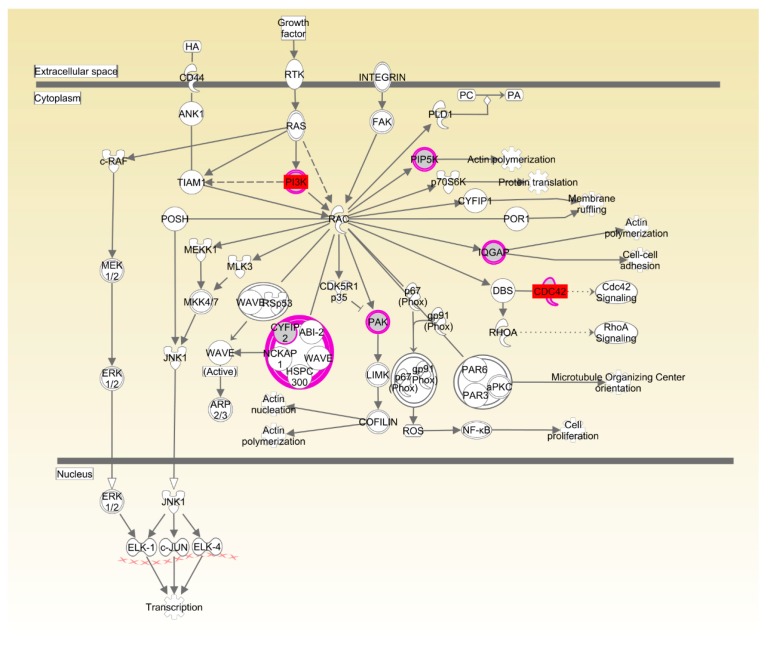
Rac signaling pathway in Cobb chickens. Molecular interaction and symbols are the same as the description in Figure 6. The red symbols show the genes that were differentially expressed in breast muscle.

**Figure 10:**
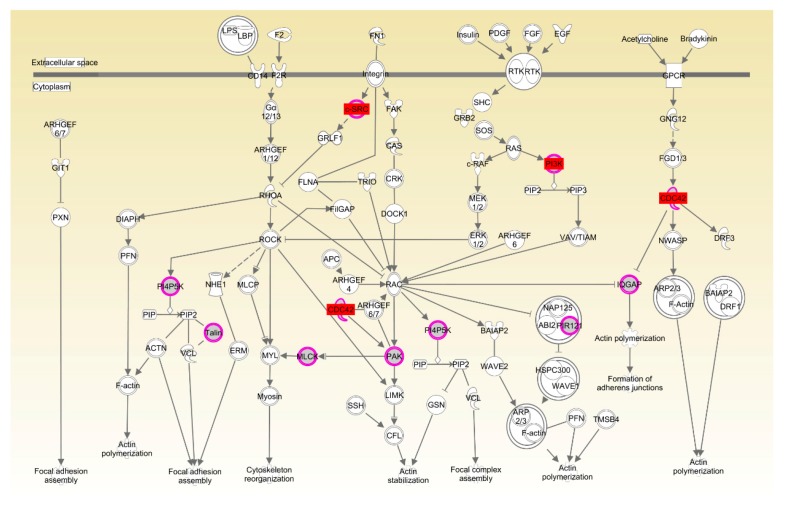
Actin cytoskeleton signaling pathway in Cobb chickens. Molecular interaction and symbols are the same as the description in Figure 6. The red symbols show the genes that were differentially expressed in breast muscle.

Again, in Cobb, the enrichment of Rac and actin cytoskeleton signaling was consistent with the important role played by organismal development for the RFI trait (Table 4, Figure 9 and Figure 10). Transcript abundance of the key genes (*CDC42*, *CSK* and *PIK3R3*) in Rac and actin cytoskeleton signaling were consistently higher (*p* < 0.01) in breast muscle of LRFI than HRFI chickens (Figure 5B).

### 3.5. SNP Validation

The genome sequencing used DNA from 16 chickens in each of the 12 pools. Nine SNPs were randomly chosen from the candidate SNPs and were subjected to verification by PCR analysis and Sanger sequencing using DNA from individual birds. The results showed (Table S6) that the average similarity was about 75% between the WGS and Sanger sequencing methods, indicating the reliability of the WGS results from the pooling strategy used here.

### 3.6. Association of the SNPs with the Breeding Value of RFI in the Cobb Population of 779

The association analysis to validate the 191 SNPs was done using the validation population of 779 offspring from the Cobb chickens making up the WGS resource (Table S7). There were 46 SNPs that were demonstrated to be significantly associated with RFI (*p* < 0.05). The details of these significant SNPs, including their positions in the genome, the nearest known genes and the *p*-values, are given in Table 5. The association between 46 SNPs genotypes and RFI was estimated. Of these, twelve SNPs, the genotypes showed significant differences. The effects of the genotypes and additive and dominance effects are shown in Table 6.

## 4. Discussion

Genetic improvement of economically-important traits, such as growth rate and feed efficiency, has occurred for about 100 years in the Cobb chickens. There has been no systematic genetic selection in the conservation population of the local Beijing-You chickens. Consistent with the current findings, more genomic variants exist in the Beijing-You than Cobb chickens, because the high intensity of genetic selection in the latter would be expected to result in genomic regions with increased homozygosity. There were numerous SNPs associated with RFI in the Beijing-You and a larger proportional difference in FCR (HRFI/LRFI = 1.2), suggesting a high potential for genetically-increasing feed efficiency in Beijing-You chickens. Obviously, FCR is already much lower in Cobb chickens, and there was less difference (HRFI/LRFI = 1.08) between the phenotypic extremes.

The two breeds examined here showed some unique characteristics, and only 127 genes associated with RFI were identified in both breeds. These represented a small proportion of the total (127/1137) for Beijing-You, but much higher (127/448) for Cobb chickens. PTEN signaling, as well as lipid and carbohydrate metabolism were only enriched in Beijing-You chickens. The PTEN/PI3K/AKT signaling pathway has been shown to regulate cell metabolism, growth and survival [16,17]. PTEN/PI3K/AKT signaling can mediate the insulin-stimulated activation of many major pathways of hepatic lipid metabolism, including lipogenesis, fatty acid β-oxidation, fatty acid esterification to triglycerides and very low density lipoprotein-triglyceride (VLDL-TG) secretion [18,19]. PTEN, a negative regulator, dephosphorylates PIP3 to inhibit the activity of PI3K/AKT signaling [20]. When the PTEN was overexpressed, insulin signaling effects were inhibited, resulting in decreased RAC-alpha serine/threonine-protein kinase (AKT) activity and solute carrier family 2, facilitated glucose transporter member 4 (GLUT4) translocation to the cell membrane, which consequently contributed to insulin resistance and progression of non-alcoholic fatty liver disease [21,22,23]. In addition to controlling the activity of signaling intermediates at the cell membrane, PI3Ks can drive gene expression programs in the nucleus. The forkhead box protein O1(FOXO) transcription factors are coupled to PI3K activity through AKT-dependent phosphorylation [24], which are considered effectors of the pathway regulating hepatic glucose production. Matsumoto et al. [25] showed that delivery of FOXO1 to mouse liver resulted in steatosis arising from increased triglyceride accumulation and decreased fatty acid oxidation. In the present study, the genes (*PHKG1*, *PIP5K1B* and *NEU3*) that participate in carbohydrate and lipid metabolism [26,27,28] were upregulated in the livers of HRFI Beijing-You chickens. Previous studies [5,29] have demonstrated that HRFI animals required more energy to maintain biological activities, such as protein turnover, stress response and muscle activity. Beijing-You chickens are characterized by high fat deposition in muscle and abdominal adipose [30]. Therefore, lipid and carbohydrate metabolism may play crucial roles in RFI through the PTEN/PI3K/AKT signaling pathway in Beijing-You chickens. 

For Cobb chickens, the results showed that feed behavior and the related pathway (CREB signaling in neurons) were enriched. Feed behavior plays a key role in feed intake, and variation in feed intake is associated with variation in RFI. The CREB transcription factor has been widely investigated as a key factor in the control of appetite and FI by the hypothalamus [31]. A previous report showed that an increase and phosphorylation of CREB in the hypothalamus, as a result of injection of NPY into the rat hypothalamus or fasting for 48 h, suggested that CREB phosphorylation might be involved in the regulation of feeding behavior [32]. Calcium/calmodulin-dependent protein kinase (CAMK) plays a critical role in activation of CREB via Ser-133 phosphorylation [33]. *PLCB4* and *CAMK4* mRNA levels were higher in the hypothalamus of HRFI than LRFI chickens. The upregulation of *CAMK4* mRNA levels may increase FI through induction of CREB phosphorylation and activation of downstream signaling pathways, and upregulation of *PLCB4* expression may affect activation of CAMKs through changes in Ca^2+^ concentrations. Therefore, the CREB signaling pathway may contribute to RFI in Cobb chickens by affecting feed behavior. Compared to the Beijing-You, the Cobb is a specialized broiler breed as a result of long-term selection for increased feed efficiency and growth rate, which may have resulted in part from changes in feed behavior and its related genetic basis. 

Organismal development as the common physiological process was enriched in both breeds. The concept of RFI was initially developed to determine differences among animals in feed conversion into body tissue. A previous study in cattle showed a variation in RFI of 37%, which was explained by tissue metabolism, protein turnover and stress response [34]. Global RNA expression in breast muscle obtained from a male broiler line phenotyped for high or low feed efficiency showed that significant differences in gene expression profiles were associated with muscle fiber development, muscle function and cytoskeletal organization [35]. In the present study, skeletal and muscular system development and function were enriched in both Beijing-You and Cobb breeds. Phenotypic measurement showed that the percentages of breast and thigh muscle were higher in LRFI than HRFI Beijing-You chickens (Table S8), suggesting that the development and function of the skeletal and muscular systems might control efficient conversion of dietary nutrients to lean tissue and growth. The canonical pathways that participated in the organismal development were also enriched in both Beijing-You and Cobb breeds. nNOS plays an important role in the regulation of many muscle functions, such as blood flow, contraction and metabolism [36]. For example, nitric oxide (NO), which is physiologically synthesized in a tightly-controlled manner by NO synthases (NOSs), directly inhibits mitochondrial respiration by interfering with electron transport chain complexes, which consequently affects skeletal muscle metabolism and stimulates glucose transport by activating upstream signaling that leads to increased production of GLUT4 [37]. Rac and actin cytoskeleton signaling has been identified in various morphogenetic events associated with tissue organization during organ development. For instance, the conserved role of paxillin in promoting signals from RAC to the actin cytoskeleton is crucial for normal tissue morphogenesis during wing and leg development of *Drosophila* [38]. Ras-related C3 botulinum toxin substrate 1 (Rac1) and Cell division control protein 42 homolog (CDC42) have also been implicated in neural development and myogenesis [39]. CSK-1 was also essential in the organization of pharyngeal muscle filaments [40]. In the present study, expression in muscle of *CDC42*, *CSK* and *PIK3R* was significantly upregulated in LRFI chickens, as compared to HRFI chickens. Therefore, all of these results showed that although there were small breed differences in the participating pathways, organismal development as the common physiological process contributed to the RFI in the two breeds.

In order to further validate the reliability of the WGS results, 191 SNPs were selected for genotyping and association analysis. The results showed that there were 46 SNPs (48 genes) significantly associated with the RFI, which demonstrated that the WGS result was reliable, and these SNPs could prove to be interesting markers for RFI. 

## 5. Conclusions

The present study provides novel insights into genomic variants related to RFI in two very distinct breeds of chicken. A total of 3746 reliable SNPs included in 1137 genes in Beijing-You chickens and 448 genes with 575 SNPs in Cobb chickens were identified as significant variant markers associated with RFI. Forty-six SNPs were significantly associated with the RFI in an independent population of 779 Cobb chickens, suggesting that the method of screening the associated SNP with WGS strategy was reasonable. Lipid and carbohydrate metabolism and the PTEN signaling pathway were enriched in the Beijing-You, while feed behavior and the CREB signaling pathway were enriched in Cobb chickens. Organismal development as the common physiological process was enriched in the two breeds. *NOS1*, *PHKG1*, *NEU3* and *PIP5K1B* were differentially expressed in Beijing-You, while *CDC42*, *CSK*, *PIK3R3*, *CAMK4* and *PLCB4* were differentially expressed in Cobb, suggesting that these might be key genes that contribute to RFI. This present study, with chickens of very distinct backgrounds and characteristics, offers important knowledge of biomarkers, candidate genes and potential biological pathways underlying RFI.

## Figures and Tables

**Table 1:** The information of the experimental population.

Breed	Experimental Population	Family No. of the Population	Birds for High and Low RFI Group	Family No. of Sub-Group	No. of DNA Pools	Average Coverage/Pool
Beijing-You	200 males	75:153 (males:females)	48 HRFI	33:40 (males:females)	3	20×
	200 females		48 LRFI	35:39 (males:females)	3	20×
Cobb	220 males	64 (males)	48 HRFI	34 (males)	3	20×
			48 LRFI	28 (males)	3	20×

For Beijing-You chickens, each pool consisted of 8 males and 8 females. For Cobb chickens, each pool consisted of 16 males. HRFI: high residual feed intake; LRFI: low residual feed intake.

**Table 2:** Performance of Beijing-You and Cobb chickens used for sequencing (*n* = 48 per group).

Breed	Measurement	LRFI	HRFI	*p*-Value
Beijing-You	RFI (g)	−239.73 ± 74.97	300.67 ± 120.98	<0.01
	DFI (g)	88.34 ± 15.11	107.28 ± 16.43	<0.01
	Initial BW (g)	820.58 ± 115.79	805.15 ± 95.36	>0.05
	Final BW (g)	1364.38 ± 254.89	1357.27 ± 218.45	>0.05
	ADG (g)	19.42 ± 5.49	19.54 ± 4.72	>0.05
	FCR	4.70 ± 0.58	5.62 ± 0.63	<0.01
Cobb	RFI (g)	−93.83 ± 7.29	104.09 ± 54.41	<0.01
	DFI (g)	141.12 ± 14.36	157.52 ± 6.11	<0.01
	Initial BW (g)	1012.71± 66.78	1018.92 ± 58.71	>0.05
	Final BW (g)	2109.06 ± 175.07	2139.08 ± 96.29	>0.05
	ADG (g)	78.31 ± 11.22	80.01 ± 5.15	>0.05
	FCR	1.82 ± 0.12	1.97 ± 0.05	<0.01

The significance of differences between LRFI and HRFI birds was determined using Student’s *t*-test. RFI: residual feed intake; DFI: daily feed intake; ADG: average daily gain over the assessed feeding period; BW: body weight; FCR: feed conversion ratio = DFI/ADG.

**Table 3:** Top five biological functions enriched in Beijing-You and Cobb chickens.

Breed	Name	*p*-Value	No. Genes
Beijing-You	Nervous system development and function	2.28 × 10^−2^–3.54 × 10^−7^	134
	Tissue development	2.52 × 10^−2^–3.54 × 10^−7^	269
	Connective tissue development and function	2.52 × 10^−2^–2.36 × 10^−5^	116
	Skeletal and muscular system development and function	2.32 × 10^−2^–2.36 × 10^−5^	111
	Organismal development	2.39 × 10^−2^–1.07 × 10^−4^	254
Cobb	Nervous system development and function	2.86 × 10^−2^–3.27 × 10^−4^	69
	Tissue morphology	2.86 × 10^−2^–3.27 × 10^−4^	47
	Behavior	2.86 × 10^−2^–8.17 × 10^−4^	9
	Connective tissue development and function	2.86 × 10^−2^–8.17 × 10^−4^	12
	Skeletal and muscular system development and function	2.86 × 10^−2^–8.17 × 10^−4^	17

A summary of results from Ingenuity^®^ Pathway Analysis (IPA) software (Ingenuity System Inc., Redwood City, CA, USA). The function in bold was specific for Cobb chickens.

**Table 4:** Top five canonical pathways enriched in Beijing-You and Cobb.

Breed	Name	*p*-Value	Overlap
Beijing-You	nNOS signaling in skeletal muscle cells	2.18 × 10^−3^	44.4%, 4/9
	All-trans-decaprenyl diphosphate biosynthesis	4.81 × 10^−3^	100.0%, 2/2
	PTEN signaling	9.73 × 10^−3^	14.3%, 13/91
	Small cell lung cancer signaling	1.09 × 10^−2^	15.9%, 10/63
	CNTF signaling	1.28 × 10^−3^	17.4%, 8/46
Cobb	CREB signaling in neurons	3.68 × 10^−3^	7.8%, 10/128
	T cell receptor signaling	6.38 × 10^−3^	9.1%, 7/77
	Neuropathic pain signaling in dorsal horn neurons	1.02 × 10^−2^	8.3%, 7/84
	Rac signaling	1.37 × 10^−2^	7.9%, 7/89
	Actin cytoskeleton signaling	1.42 × 10^−2^	8.3%, 7/84

A summary of result from Ingenuity^®^ IPA software. PTEN: phosphatase and tensin homolog; CNTF: ciliary neurotrophic factor; CREB: cAMP responsive element binding protein; Rac: Ras-related C3 botulinum toxin substrate.

**Table 5:** SNPs significantly associated with residual feed intake (RFI) (*p* < 0.05) in Cobb chickens.

GGA ^a^	SNP	Position	*p*-Value	Candidate/Nearest Gene	Location ^b^
13	rs317270265	12, 853, 825	8.12 × 10^−6^	*CANX*	Downstream
21	rs14286155	6, 350, 615	3.06 × 10^−5^	*CDC42*	Intron
2	rs315791208	23, 470, 408	4.36 × 10^−5^	*GNG11*	Intron
13	rs317965159	10, 726, 350	0.000684	*CYFIP2*	Intron
2	rs315951802	56, 946, 139	0.001189	*ATP9B*	Intron
15	-	7, 489, 912	0.001298	*MN1*	Extron
1	rs15213482	25, 118, 696	0.001437	*TES*	Intron
Z	rs312714432	7, 564, 759	0.002362	*FAM219A*	Intron
1	rs14080181	468, 436	0.003409	*PPP6R2*	Intron
1	rs318069175	25, 124, 828	0.003993	*TES*	Intron
15	rs15775634	7, 501, 967	0.004145	*MN1*	Intron
Z	-	46, 332, 884	0.005222	*STARD4*	Upstream
15	rs314540962	7, 528, 893	0.005293	*PITPNB*	Downstream
1	rs315382419	130, 499, 091	0.005896	*GABRG3*	Intron
1	rs317493245	130, 499, 100	0.006448	*GABRG3*	Intron
6	rs14588839	27, 221, 456	0.007046	*CCDC186*	Intron
18	-	4, 638, 932	0.007055	*UNC13D*, *ENSGALG00000002278*	Downstream
4	-	66, 157, 841	0.008525	*TXK*, *TEC*	Upstream
6	-	20, 250, 604	0.008611	*HHEX*	Downstream
10	rs316109660	1, 504, 159	0.008684	*NEO*	Intron
7	rs316483815	26, 455, 736	0.009332	*ADCY5*	Intron
27	rs14301531	1, 697, 235	0.011689	*PSMC5*, *FTSJ3*, *ENSGALG00000000293*, *SMARCD2*	Upstream, Extron, Downstream, Downstream
7	rs15872356	26, 615, 279	0.015109	*ADCY5*	Intron
7	rs315234262	26, 817, 253	0.019031	*MYLK*	Intron
Z	-	6, 813, 606	0.019644	*KIAA1328*	Intron
20	rs13633836	8, 919, 435	0.020108	*HAR1A*	Upstream
Z	-	6, 847, 225	0.02043	*KIAA1328*	Intron
6	rs312986238	27, 242, 319	0.020633	*TDRD1*	Upstream
5	-	16, 429, 960	0.022312	*TPCN2*	Intron
2	rs15931222	28, 639, 260	0.024226	*BZW2*	Intron
4	rs315081661	12, 239, 357	0.024332	*USP12P1*	Downstream
4	-	36, 460, 098	0.024731	*GRID2*	Intron
4	rs318158632	5, 122, 558	0.026111	*NOX1*, *ENSGALG00000006637*, *ENSGALG00000020303*	Upstream, Intron, Downstream
26	rs14300622	4, 106 ,086	0.027252	*SCUBE3*	Intron
15	rs14092096	7, 493, 037	0.029197	*MN1*	Intron
3	rs15269609	8, 547, 001	0.030645	*MSH2*	Intron
4	rs15588679	56, 165, 356	0.033439	*CAMK2D*	Intron
2	rs313915675	39, 376, 196	0.034241	*CMC1*	Intron
4	rs16400807	45, 122, 907	0.034602	*NUDT9*, *ENSGALG00000010963*	Upstream
6	-	20, 691, 988	0.039963	*BLNK*	Intron
4	rs13523480	56, 160, 482	0.041005	*CAMK2D*	Intron
2	-	145, 118, 378	0.042982	*TRAPPC9*	Intron
4	-	67, 677, 262	0.045337	*GRXCR1*	Intron
2	rs15067942	16, 967, 429	0.048495	*KIAA1217*	Intron
3	rs14316028	8, 484, 254	0.049147	*FAM179A*, *TEC*	Upstream
13	rs313110716	3, 909, 846	0.049643	*SLIT3*	Intron

^a^ Chicken chromosome. ^b^ The location of SNPs on genes. Genes in bold involved in the key pathways for the RFI.

**Table 6:** Effect of the genotypes on RFI in Cobb chickens.

SNP	GGA	Position	*N*	Genotype	LSM ± SD	Additive Effect	Dominance Effect
rs15213482	chr1	25, 118, 696	125	AA	35.02 ± 7.65 ^B^	14.41	−10.70
			330	AG	9.91 ± 4.69 ^A^		
			320	GG	6.20 ± 4.76 ^A^		
rs318069175	chr1	25, 124, 828	318	AA	6.08 ± 4.79 ^A^	−12.95	−8.31
			329	AG	10.72 ± 4.71 ^A^		
			127	GG	31.98 ± 7.63 ^B^		
-	chr2	145, 118, 378	276	CC	1.57 ± 5.14 ^A^	−12.65	0.17
			357	TC	14.39 ± 4.50 ^AB^		
			141	TT	26.86 ± 7.18 ^B^		
rs315791208	chr2	23, 470, 408	166	AA	−3.58 ± 6.62 ^A^	−8.82	12.43
			380	AG	17.67 ± 4.36 ^B^		
			230	GG	14.05 ± 5.61 ^B^		
rs313915675	chr2	39, 376, 196	623	AA	9.67 ± 3.44 ^a^	10.14	29.01
			123	AC	28.55 ± 7.72 ^b^		
			27	CC	−10.60 ± 16.47 ^a^		
rs315951802	chr2	56, 946, 139	64	AA	−1.63 ± 10.68 ^a^	−10.64	−3.53
			293	AG	5.49 ± 5.00 ^a^		
			417	GG	19.66 ± 4.2 ^b^		
rs318158632	chr4	5, 122, 558	249	AA	14.44 ± 5.41 ^b^	8.36	11.93
			362	AG	18.01 ± 4.50 ^b^		
			167	GG	−2.29 ± 6.59 ^a^		
rs315081661	chr4	12, 239, 357	215	CC	15.95 ± 5.82 ^b^	8.83	10.09
			386	TC	17.21 ± 4.35 ^b^		
			175	TT	−1.71 ± 6.45 ^a^		
-	chr5	16, 429, 960	246	AA	23.49 ± 5.46 ^b^	10.08	−5.28
			363	AT	8.13 ± 4.50 ^a^		
			164	TT	3.33 ± 6.70 ^a^		
rs316109660	chr10	1, 504, 159	50	AA	32.29 ± 12.03 ^b^	7.97	−20.56
			325	AG	3.76 ± 4.72 ^a^		
			400	GG	16.34 ± 4.27 ^b^		
-	chr18	4, 638, 932	223	CC	23.67 ± 5.72 ^B^	10.90	−1.90
			361	CG	10.86 ± 4.48 ^AB^		
			193	GG	1.86 ± 6.16 ^A^		
-	chrZ	46, 332, 884	133	AA	12.64 ± 7.68 ^AB^	−2.78	−20.25
			145	AG	−4.82 ± 7.53 ^A^		
			497	GG	18.21 ± 3.90 ^B^		

*N*: number of genotype records. ^a,b^ Means in the same column with different superscripts significantly differ at *p* < 0.05. ^A,B^ Means in the same column with different superscripts significantly differ at *p* < 0.01.

## Supplementary Materials

The following are available online at http://www.mdpi.com/2073-4425/9/2/57/s1. Table S1. Primer information used in amplification for Sanger sequencing (DOCX). Table S2. Oligonucleotide PCR primers (DOCX). Table S3. Results of whole genome sequencing and assembly (DOCX). Table S4. Candidate SNPs related to RFI in Beijing-You chickens (XLSX). Table S5. Candidate SNPs related to RFI in Cobb chickens (XLSX). Table S6. Verification of nine SNPs using PCR analysis and Sanger sequencing (DOCX). Table S7. The result of the association analysis between RFI and candidate SNPs (XLSX). Table S8. Carcass traits of the Beijing-You chickens used for sequencing (DOCX). Figure S1. Lipid and carbohydrate metabolism networks in Beijing-You chickens (DOCX).
