# Supplementary material for: Exploring Genomic Variants Related to Residual Feed Intake in Local and Commercial Chickens by Whole Genomic Resequencing
Source: Genes (Basel). 2018 Jan 24;9(2):57. doi: 10.3390/genes9020057 (PMC5852553; doi:10.3390/genes9020057)
Supplement: Supplementary file 1 [file genes-09-00057-s001.zip › genes-238553-Supplementary Material/Supplementary Material/S1Figure.docx]

**
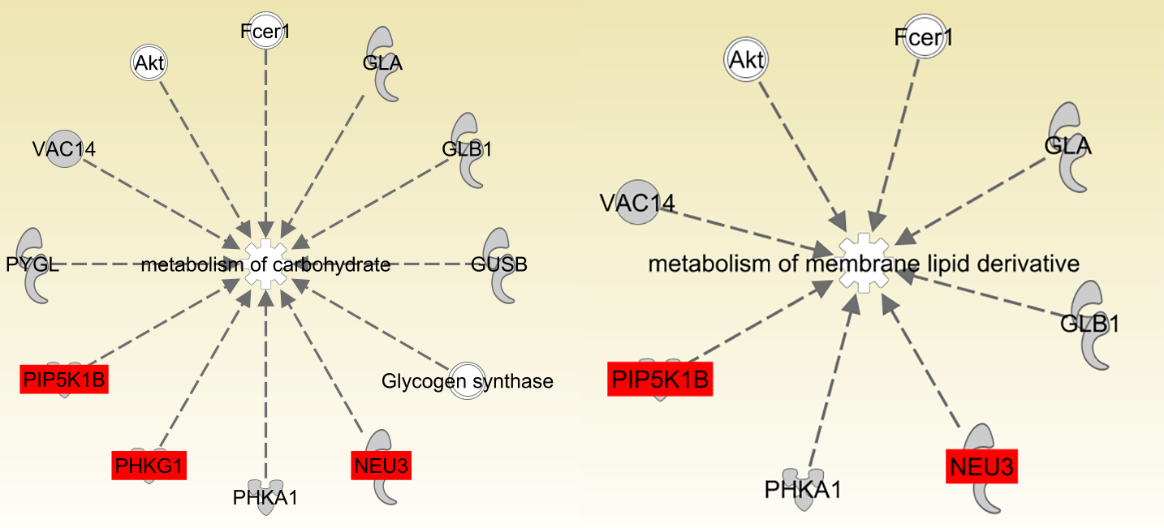
**

**S1 Figure. The gene network with functions in lipid and carbohydrate metabolism in Beijing-You.** The genes marked with red were differentially expressed genes in liver from qRT-PCR analysis.
