# Supplementary material for: Exploring Genomic Variants Related to Residual Feed Intake in Local and Commercial Chickens by Whole Genomic Resequencing
Source: Genes (Basel). 2018 Jan 24;9(2):57. doi: 10.3390/genes9020057 (PMC5852553; doi:10.3390/genes9020057)
Supplement: Supplementary file 1 [file genes-09-00057-s001.zip › genes-238553-Supplementary Material/Supplementary Material/Table S1.docx]

Table S1 The primers information using in amplicon for Sanger sequencing

| SNP | Primer information |
| --- | --- |
| rs15538501 | 5'TTACTGAGCGCAAAGAGAGG3' |
|  | 5'TTAGGTCATGGTTGGACTCA3' |
| rs314542908 | 5'GTGTTTTGGACTCCTGGGAA3' |
|  | 5'GTGCAGAATGGATGGATATG3' |
| rs13641001 | 5'AATCTGTGCTGCGCTTTGTA3' |
|  | 5'AAGCAGTGGGTTCGTGAGTT3' |
| rs313744404 | 5'ATGCTGTGGGTTCCCGTTTC3' |
|  | 5'GCACAAAGCCCTCTCACTAA3' |
| rs313650192 | 5'TTCATGCTGATTGCTTCTTC3' |
|  | 5'GTGCTCACCACATCAGGATA3' |
| rs313744154 | 5'AAGGGACGCTTGGTGTCATA3' |
|  | 5'GGGAGATGCAAGCTAGGAAG3' |
| rs313145934 | 5'GCAGTGTGGCTCAGTGTATG3' |
|  | 5'CATTTGCGGAGGTGTCAGTT3' |
| rs317486776 | 5'TGGCAGATCATAGCTTCAGA3' |
|  | 5'TAGAGTAGCAGGCAAGATCC3' |
| rs312686693 | 5'GCACAGGCATAACGTCAGT3' |
|  | 5'AACACAGCCCAGACTTCAAT3' |
