# Supplementary material for: Exploring Genomic Variants Related to Residual Feed Intake in Local and Commercial Chickens by Whole Genomic Resequencing
Source: Genes (Basel). 2018 Jan 24;9(2):57. doi: 10.3390/genes9020057 (PMC5852553; doi:10.3390/genes9020057)
Supplement: Supplementary file 1 [file genes-09-00057-s001.zip › genes-238553-Supplementary Material/Supplementary Material/Table S2.docx]

Table S2 Oligonucleotide PCR primers.

| Gene | Accession number ^a^ | Primer sequence 5’→3’ | Orientation | Product size (bp) |
| --- | --- | --- | --- | --- |
| CDC42 | NM_205048 | AACAGTGATGATTGGAGGAG | Forward | 294 |
|  |  | TCTGGAGTTATGGGCTTC | Reverse |  |
| FGFR4 | XM_015293863.1 | AGCACATCGAGGTGAACGG | Forward | 190 |
|  |  | AGCCACGCTGACTGGTAGG | Reverse |  |
| NOS1 | XM_015294873.1 | AGCCCTAAGTCCAGTCGC | Forward | 146 |
|  |  | TCCTCAAGCCTGTCAATCA | Reverse |  |
| PIK3R3 | XM_004936762.2 | GTTATGCCTGTTCTGTGGT | Forward | 168 |
|  |  | CATTGAGGGAATCGTTGT | Reverse |  |
| PIP5K1B | NM_001031422.1 | CTGGAAAGCCAAATGAAG | Forward | 168 |
|  |  | CACTGGGTAAGAAGACACT | Reverse |  |
| PHKG1 | NM_001006217.2 | GGGCTACGGGAAAGAAGT | Forward | 179 |
|  |  | TCAGGTCCTTGACGGTGT | Reverse |  |
| CSK | NM_205425.1 | TCGGGCTGACAAAGGAAG | Forward | 170 |
|  |  | TCTCGGATAAGGCACTCG | Reverse |  |
| NEU3 | NM_001319017.1 | GCCCGCACTCCTTCATCTT | Forward | 129 |
|  |  | GCAGCGTCCCTCCATCCTT | Reverse |  |
| CAMK4 | NM_001034813.1 | TCCTTCTTCGCCTTTCAC | Forward | 183 |
|  |  | CAACCGCCTCCAGTATCT | Reverse |  |
| PLCB4 | NM_001199435.1 | AATGGGTAGAAGGACTGG | Forward | 142 |
|  |  | CTGGTAATGCTCCGAACT | Reverse |  |

^a^ Accession number refer to Genbank (NCBI).
