# Supplementary material for: Exploring Genomic Variants Related to Residual Feed Intake in Local and Commercial Chickens by Whole Genomic Resequencing
Source: Genes (Basel). 2018 Jan 24;9(2):57. doi: 10.3390/genes9020057 (PMC5852553; doi:10.3390/genes9020057)
Supplement: Supplementary file 1 [file genes-09-00057-s001.zip › genes-238553-Supplementary Material/Supplementary Material/Table S3.docx]

Table S3. Results of whole genome sequencing and assembly.

| Sample | Raw Reads Number | Clean Reads Number | Clean Reads Rate (%) | Aligned Reads Number | Coverage  (X) | Number of SNP |
| --- | --- | --- | --- | --- | --- | --- |
| CB(L1)^1^ | 163,897,774 | 158,806,790 | 96.89 | 146,070,818 | 18.96 | 8,352,008 |
| CB(L2) | 173,456,110 | 167,863,062 | 96.78 | 154,573,426 | 20.04 | 8,352,008 |
| CB(L3) | 165,303,990 | 160,230,310 | 96.93 | 147,767,897 | 19.13 | 8,352,008 |
| CB(H1)^2^ | 167,559,048 | 162,462,800 | 96.96 | 148,287,306 | 19.40 | 8,372,769 |
| CB(H2) | 168,043,048 | 161,995,526 | 96.4 | 147,920,055 | 19.34 | 8,372,769 |
| CB(H3) | 168,111,306 | 162,393,800 | 96.6 | 146,846,737 | 19.39 | 8,372,769 |
| BJY(L1)^3^ | 170,711,080 | 164,643,296 | 96.45 | 150,782,532 | 19.66 | 8,505,214 |
| BJY(L2) | 152,123,828 | 146,180,018 | 96.09 | 133,633,094 | 17.45 | 8,505,214 |
| BJY(L3) | 169,161,464 | 163,774,498 | 96.82 | 149,151,483 | 19.55 | 8,505,214 |
| BJY(H1)^4^ | 164,816,766 | 160,026,212 | 97.09 | 145,470,898 | 19.11 | 8,479,041 |
| BJY(H2) | 155,480,592 | 150,405,688 | 96.74 | 138,258,079 | 17.96 | 8,479,041 |
| BJY(H3) | 161,175,790 | 157,160,498 | 97.51 | 143,966,175 | 18.76 | 8,479,041 |

^1^CB(L1-3), Cobb LRFI chickens; ^2^CB(H1-3), Cobb HRFI chickens; ^3^BJY(L1-3), Beijing-You LRFI chickens; ^4^BJY(H1-3), Beijing-You HRFI chickens.
