# Supplementary material for: Exploring Genomic Variants Related to Residual Feed Intake in Local and Commercial Chickens by Whole Genomic Resequencing
Source: Genes (Basel). 2018 Jan 24;9(2):57. doi: 10.3390/genes9020057 (PMC5852553; doi:10.3390/genes9020057)
Supplement: Supplementary file 1 [file genes-09-00057-s001.zip › genes-238553-Supplementary Material/Supplementary Material/Table S6.docx]

Table S6. Verification of nine SNPs using PCR analysis and Sanger sequencing.

| Chr. | Ref pos | Ref base | Called base | Impact | SNP% WGS | SNP | SNP% validation |
| --- | --- | --- | --- | --- | --- | --- | --- |
| 1 | 184952082 | C | A | LRFI | 100% | rs15538501 | 87% |
| 2 | 80054883 | T | G | LRFI | 25% | rs314542908 | 44% |
| 4 | 2941781 | G | T | LRFI | 100% | rs13641001 | 88% |
| 5 | 57849939 | G | C | LRFI | 100% | rs313744404 | 88% |
| Z | 645634 | T | C | LRFI | 50% | rs313650192 | 69% |
| 10 | 19486516 | C | A | LRFI | 33% | rs313145934 | 63% |
| 7 | 4192517 | T | C | HRFI | 63% | rs317486776 | 94% |
| 20 | 10519119 | A | G | HRFI | 100% | rs312686693 | 88% |
| 2 | 34267466 | G | C | HRFI | 100% | rs313744154 | 100% |
