# Supplementary material for: Exploring Genomic Variants Related to Residual Feed Intake in Local and Commercial Chickens by Whole Genomic Resequencing
Source: Genes (Basel). 2018 Jan 24;9(2):57. doi: 10.3390/genes9020057 (PMC5852553; doi:10.3390/genes9020057)
Supplement: Supplementary file 1 [file genes-09-00057-s001.zip › genes-238553-Supplementary Material/Supplementary Material/Table S8.docx]

**Table S8. Carcass traits of Beijing-You used for sequencing**

| **Measurement** | **LRFI** | **HRFI** | ***p* value** |
| --- | --- | --- | --- |
| Breast muscle percentage (%) | 15.44 ± 2.57 | 13.42 ± 2.38 | < 0.01 |
| Thigh muscle percentage (%) | 20.58 ± 1.64 | 19.51 ± 1.65 | < 0.05 |
